# ARGUE-TIRA: attention-based trait interaction reasoning for holistic and analytic automated essay scoring

**DOI:** 10.3389/frai.2026.1895359

**Published:** 2026-09-18

**Authors:** Areej Alhothali

**Affiliations:** Department of Computer Science, Faculty of Computing and Information Technology, King Abdulaziz University, Jeddah, Saudi Arabia

**Keywords:** analytic rubric scoring, automatic essay scoring, educational natural language processing, holistic scoring, multi-task learning, transformer models

## Abstract

Automated Essay Scoring (AES) has emerged as an important application of natural language processing providing scalable and consistent evaluation of written responses. These assessments can be conducted using holistic or analytic evaluation approaches. Recent studies have heavily focused on utilizing deep learning models and particularly transformer-based models in holistic essay scoring, while accurately modeling the multi-dimensional analytical scoring remains a challenging problem. Despite their strong performance in holistic essay scoring, most existing approaches work as black-box predictors and do not explicitly model the reasoning process underlying analytic rubric-based evaluation. In this paper, we propose ARGUE-TIRA, a multi-task framework for joint holistic and analytic essay scoring. The proposed framework first predicts multiple analytic writing traits and subsequently applies a Trait Interaction Reasoning Attention (TIRA) to explicitly model dependencies among rubric dimensions before generating the final holistic score. Unlike conventional multi-task AES approaches that independently predict analytic traits, the proposed reasoning mechanism captures inter-trait relationships through structured self-attention reasoning, improving both scoring consistency and interpretability. Experiments conducted on the ASAP and ASAP++ datasets demonstrate that ARGUE-TIRA achieves competitive holistic performance and strong analytic trait-scoring performance. The framework obtains an average holistic QWK of 0.762 on ASAP and the highest average analytic QWK of 0.7424 on ASAP++. Furthermore, it achieves the best performance in seven of the eight evaluated analytic traits, including content, organization, word choice, sentence fluency, conventions, prompt adherence, and language quality.

## 1 Introduction

Automated Essay Scoring (AES) is one of the important applications of natural language processing (NLP), which aims to provide scalable and consistent evaluation of written text. Due to the emerging use of online learning platforms and large-scale educational assessments, AES systems are widely used to support essay evaluation while reducing the workload of human graders. Despite significant progress made in recent years in deep learning and NLP applications, automatically scoring essays is a challenging problem due to the complexity of natural language, diversity of writing styles and the inherently multi-dimensional nature of writing quality.

AES approaches have evolved from handcrafted linguistic and statistical features (Page, 1966; Attali and Burstein, 2006; Rudner and Liang, 2002) to deep learning and transformer-based architectures capable of contextualized representation learning (Taghipour and Ng, 2016; Alikaniotis et al., 2016; Devlin et al., 2019). Early-stage methods primarily focused on surface-level characteristics such as grammar usage, vocabulary richness, and syntactic patterns. Despite their moderate performance, traditional machine learning approaches were limited in their ability to capture deeper semantic relationships, discourse structure, and contextual dependencies within essays (Shermis and Burstein, 2013). Deep learning, particularly transformer-based architectures, has significantly improved AES performance by enabling contextualized representation learning (Devlin et al., 2019).

However, most transformer-based AES systems have primarily focused on holistic scoring, where a single overall score is assigned to the entire essay (Taghipour and Ng, 2016; Misgna et al., 2024; Uto, 2021). In contrast, human raters often employ a rubric-based analytic assessment process when evaluating written essays. This process involves individually assessing multiple writing dimensions, including content quality, organization, language use, conventions, and prompt adherence, before combining these intermediate ratings into a final holistic score (Weigle, 2002; Attali and Burstein, 2006). Earlier AES models typically predict holistic or analytic scores without explicitly reasoning over the dependencies among rubric traits, thereby limiting the transparency of the scoring process (Mathias and Bhattacharyya, 2020).

Furthermore, benchmark datasets such as the ASAP++ dataset contain heterogeneous rubric structures, where different prompts are assigned to different analytic traits and scoring criteria. This variability presents further difficulties for the development of AES systems that can adapt to prompt-specific evaluation requirements.

Another major limitation of current AES models is that they operate primarily as predictive regression or classification models without explicitly considering interactions among analytic writing dimensions. However, in practice, writing traits are often interdependent. For instance, organization has a significant impact on sentence fluency, while prompt adherence and language use are strongly related to content quality. Ignoring these dependencies may limit the model's ability to capture the complex reasoning process underlying human essay assessment. Without these dependencies, the model might not be able to reflect the multiple layers of reasoning that might occur during human essay grading.

To address these limitations, we propose ARGUE-TIRA, a multi-task framework for joint holistic and analytic essay scoring. The proposed framework models holistic and analytic scoring jointly in a unified architecture. In particular, a transformer-based encoder is employed for generating contextual essay representations followed by an intermediate trait prediction module that predicts various dimensions of writing traits. Subsequently, a Trait Interaction Reasoning Attention (TIRA) is introduced as a self-attentive reasoning module to explicitly model dependencies among analytic traits and capture interactions among rubric dimensions before generating the final holistic score. The proposed reasoning mechanism allows the model to learn structured relations among writing dimensions, which makes the model's decisions more coherent and interpretable, unlike conventional multi-task approaches, which predict analytic traits independently.

Furthermore, the proposed model is capable of accommodating a variety of rubric structures, selectively activating prompt-relevant analytic traits, and facilitating generalization over a wide range of essay prompts and scoring rubrics. We evaluate the proposed framework on the ASAP dataset and ASAP++, an extended version of ASAP augmented with analytic trait annotations. Experimental results demonstrate that ARGUE-TIRA achieves competitive holistic performance and superior analytic trait-scoring performance compared with the evaluated baselines. Detailed prompt-level and trait-level analyses further demonstrate the effectiveness of explicit trait interaction reasoning in improving scoring consistency and interpretability. An overview of the proposed ARGUE-TIRA framework is presented in Figure 1.

**Figure 1:**
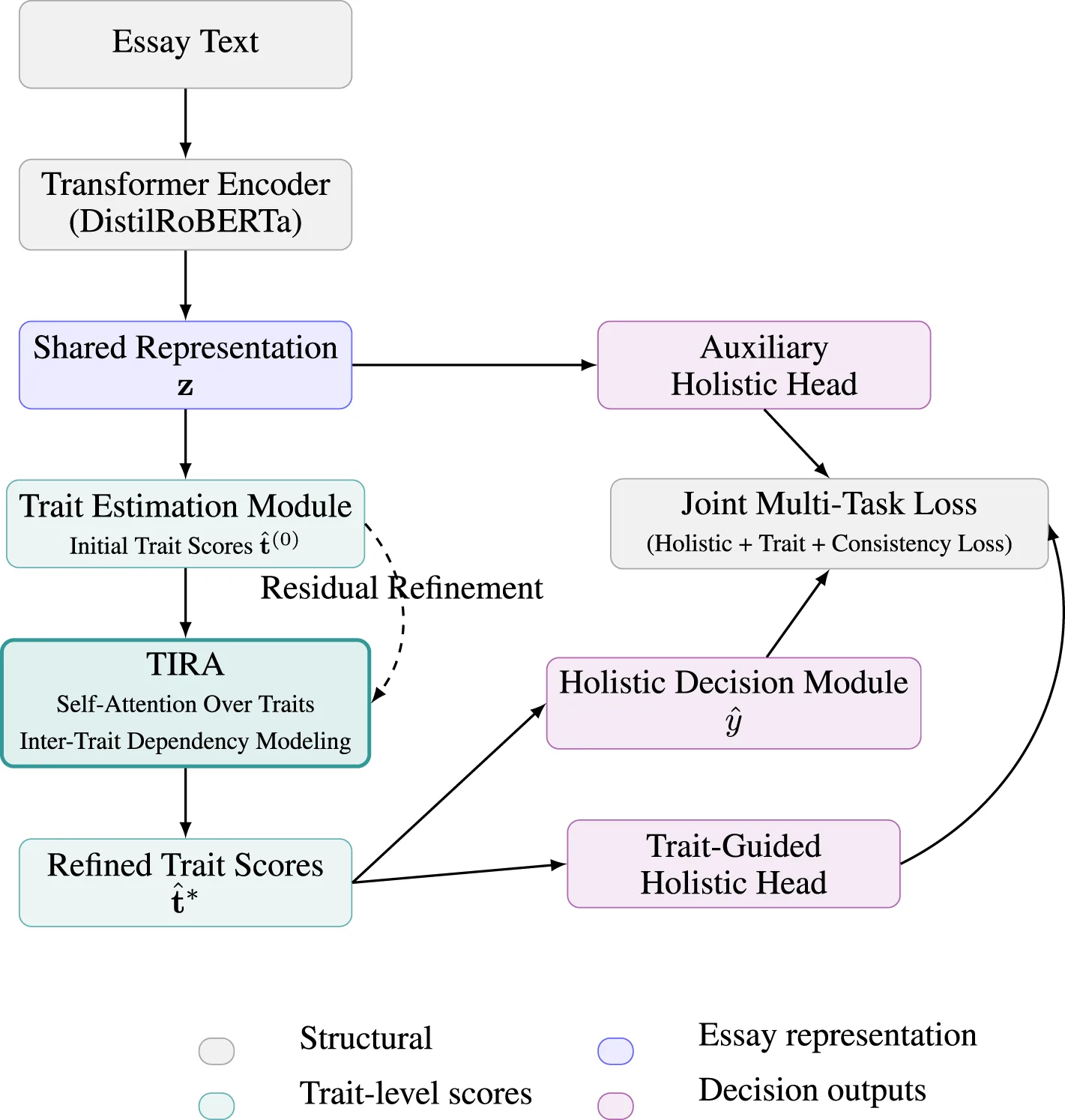
Overview of the proposed ARGUE-TIRA framework. The essay is first encoded using DistilRoBERTa to obtain a shared representation **z** (blue). Initial analytic trait scores (teal) are generated and subsequently refined through TIRA, which models inter-trait dependencies via self-attention. The refined trait scores are integrated with **z** to produce holistic decision outputs (violet) through complementary scoring heads, optimized jointly against the multi-task loss (gray).

The main contributions of this work are summarized as follows:

- We propose ARGUE-TIRA, a multi-task framework that jointly models holistic and analytic essay scoring through explicit trait-interaction reasoning.
- We introduce a Trait Interaction Reasoning Attention (TIRA), a self-attentive reasoning mechanism that explicitly captures dependencies among analytic writing traits.
- We develop a prompt-aware scoring framework capable of handling heterogeneous rubric structures across essay prompts.
- We perform extensive experiments and analysis at the overall, prompt, and trait levels, which illustrate the benefits of explicit trait interaction reasoning for enhancing AES performance and interpretability.

## 2 Related work

Automatic Essay Scoring (AES) has received substantial research interest as a major application of Natural Language Processing (NLP) in educational assessment. The classical handcrafted feature engineering techniques have progressed over time to deep learning architectures, transformer-based models, graph neural networks, and recently Large Language Model (LLM) based Frameworks (Dikli, 2006; Shermis and Burstein, 2013; Taghipour and Ng, 2016; Alikaniotis et al., 2016; Devlin et al., 2019). Current research increasingly focuses on holistic and analytic trait scoring, explainability, cross-prompt transferability, coherence modeling, and generation of educational feedback.

Early AES systems relied heavily on handcrafted linguistic and statistical features combined with classical machine learning methods such as regression models, decision trees, and Support Vector Machines (SVMs) (Page, 1966; Attali and Burstein, 2006; Rudner and Liang, 2002). These methods focused primarily on surface-level writing characteristics, including grammar usage, vocabulary richness, spelling, and syntactic complexity. Such methods were moderately successful, but were less successful at capturing deeper semantic relationships, contextual dependencies, discourse coherence and argumentative structure (Shermis and Burstein, 2013).

With the advent of deep learning, AES research made great progress with the automatic extraction of contextual and semantic representations from essay text. Recurrent neural networks (RNNs), especially long short-term memory (LSTM) and bidirectional LSTM (BiLSTM), gained popular use for modeling sequential dependencies in essays (Taghipour and Ng, 2016). Alikaniotis et al. (2016) introduced one of the first neural AES frameworks that learns distributed essay representations without the need for extensive handcrafted features. Later, attention mechanisms were integrated into neural architectures to better identify important components of the essay and information at the discourse-level (Dong et al., 2017). Several studies also explored hierarchical and hybrid deep learning architectures, including CNN-based and CNN–RNN hybrid frameworks, to jointly capture local semantic patterns and long-range contextual dependencies (Mohsin et al., 2025; Wang, 2024). SkipFlow networks further improved coherence modeling by explicitly incorporating neural coherence features into essay representations (Tay et al., 2018).

More recently, transformer-based architectures have become dominant in AES research due to their ability to capture long-range contextual dependencies using self-attention mechanisms. BERT and related transformer models provide contextualized representations that significantly improve scoring accuracy, robustness, and cross-prompt generalization (Devlin et al., 2019). Comparative analyses further indicate that encoder-based transformer architectures provide an effective balance between performance and computational efficiency for supervised AES tasks and prompt-independent scoring settings (Barbosa et al., 2025).

A major recent research direction focuses on trait-based and multi-task AES systems, where models jointly predict holistic scores together with fine-grained analytic traits such as content, organization, word choice, sentence fluency, conventions, prompt adherence, coherence, narrativity, and relevance. Cho et al. (2024) introduced the DualBERT-Trans-CNN framework that uses dual-scale BERT representations in a multi-task framework for simultaneous holistic and analytic scoring. Chen et al. (2024) also presented a Multi-Task Automated Assessment (MTAA) framework to model multiple essay quality dimensions jointly by considering orthogonality constraints and adaptive learning strategies. Ridley et al. (2021) proposed a multi-task model using the trait-attention design that could predict both holistic and trait-level scores, and improve cross-prompt transferability for unseen prompts.

Cross-prompt and cross-topic adaptation have also emerged as critical research challenges in AES. Chen and Li (2023) proposed Prompt-Mapping Contrastive Learning (PMAES) to use contrastive learning to align the representations of the source and target prompts to enhance transferability of features across prompts. To address the issue of domain generalization in cross-prompt AES, Zhang et al. (2025b) introduced Category Adversarial Joint Learning (CAJL), which combines adversarial learning with joint regression and classification objectives. Similarly, Zhang et al. (2025c) introduced the ATOP framework, where both the topic-shared and topic-specific features of the essay are jointly modeled through adversarial topic-aware prompt tuning. Chen et al. (2025) developed MOOSE, a combination-of-experts system that combines essay quality, essay comparison and prompt relevance experts for powerful cross-prompt and multi-trait scores.

In recent years a number of studies have also examined coherence-aware, relevance-aware and graph-based approaches to AES. Pan et al. (2025) introduced a Multi-Perspective Coherence Assessment (MPCA) framework, which utilizes attention mechanisms and lightweight CNNs in order to model semantic consistency, discourse coherence, and local coherence in a joint model. Albatarni et al. (2024) proposed a dense retrieval framework for scoring the relevance and adherence to prompts with Contriever embeddings. Attention has also been paid to Graph-based AES approaches. Aljuaid et al. (2025) used Transformer encoders, Graph Convolutional Networks and ensemble learning to produce holistic essay scoring. Kato (2025) introduced a model with Graph Attention Network (GAT) to model the relation between argumentative and logical sentences in an essay and boost the robustness and the correctness of the scores in the presence of imperfect argumentative structure.

Recently, the development of Large Language Models (LLMs) has shifted the paradigm of AES from supervised learning for specific tasks to pre-trained general language models that perform few-shot and zero-shot essay evaluation (Brown et al., 2020; OpenAI, 2023). Unlike traditional AES methods, LLM-based approaches often rely on prompt engineering rather than extensive task-specific training. ElMassry and Zaki (2025) investigated GPT-4.1 using systematic DSPy prompting strategies and achieved significant improvements in the accuracy of AES via the optimization of these prompts. Mansour et al. (2024) evaluated ChatGPT and Llama models for both holistic and trait-level scoring on ASAP, showing promising feedback-generation capabilities despite lower prediction accuracy compared with specialized AES systems. Winarta et al. (2024) proposed an atomic evaluation framework, which breaks down essays into small components aligned with the rubric for explainable LLM-based scoring. Chu et al. (2025) proposed a technique called RMTS that combines LLM generated trait-wise rationales with smaller scoring models to improve interpretability and reliability in multi-trait AES.

Despite these advances, several challenges remain. Many AES systems still treat essays as flat token sequences without explicitly modeling hierarchical discourse structures or long-range argumentative relationships. Transformer-based and LLM-based models also remain vulnerable to adversarial manipulations and prompt sensitivity, which can lead to unreliable or inflated scores (Silveira et al., 2025). Additional concerns include bias, interpretability, computational cost, and limited generalization across prompts, domains, and languages (Bommasani et al., 2021; Wei et al., 2022). Overall, recent AES research demonstrates a strong transition toward transformer-based multi-task architectures, graph reasoning frameworks, trait-aware scoring systems, and explainable LLM-driven evaluation models, while highlighting the need for more robust, interpretable, and generalizable AES frameworks.

## 3 Dataset

The proposed model uses the Automated Student Assessment Prize (ASAP) dataset and its analytic extension, ASAP++ (Shermis, 2014; Mathias and Bhattacharyya, 2018). ASAP was released as part of the 2012 Kaggle Automated Student Assessment Prize competition and contains 12,978 English essays written by students in Grades 7–10 in response to eight writing prompts. The prompts represent three writing conditions: persuasive or argumentative writing (Prompts 1 and 2), source-dependent responses (Prompts 3–6), and narrative writing (Prompts 7 and 8). Consequently, they differ in their communicative objectives, expected essay lengths, scoring ranges, and applicable rubric dimensions.

ASAP provides holistic scores for all eight prompts and includes original analytic rubric scores for the two narrative prompts. ASAP++ extends the resource by supplying analytic annotations for Prompts 1–6 (Mathias and Bhattacharyya, 2018). Although the complete ASAP/ASAP++ resource contains eight prompts, the present experiments use Prompts 1–6, comprising 10,686 essays, because these are the prompts for which ASAP++ provides the additional analytic annotations. The composition of the ASAP and ASAP++ datasets is summarized in Table 1.

**Table 1:** Composition of the ASAP and ASAP++ datasets, compiled from Mathias and Bhattacharyya (2018); Shermis (2014).

Prompt	Writing type	Essays	Holistic score	Analytic traits
1	Persuasive/argumentative	1,783	2–12	Content, organization, word choice, sentence fluency, conventions
2	Persuasive/argumentative	1,800	1–6	Content, organization, word choice, sentence fluency, conventions
3	Source-dependent	1,726	0–3	Content, prompt adherence, language, narrativity
4	Source-dependent	1,772	0–3	Content, prompt adherence, language, narrativity
5	Source-dependent	1,805	0–4	Content, prompt adherence, language, narrativity
6	Source-dependent	1,800	0–4	Content, prompt adherence, language, narrativity
7	Narrative	1,569	0–30	Content, organization, style, conventions
8	Narrative	723	0–60	Content, organization, voice, word choice, sentence fluency, conventions

The experimental subset covers eight dimensions: Content, Organization, Word Choice, Sentence Fluency, Conventions, Prompt Adherence, Language, and Narrativity. Content evaluates the relevance and development of the ideas required by a particular prompt; Organization concerns the structure and logical sequencing of those ideas; Word Choice evaluates the appropriateness and effectiveness of the vocabulary; Sentence Fluency concerns sentence quality and readability; and Conventions cover spelling, punctuation, capitalization, grammar, and related writing mechanics. Prompt Adherence measures how directly a response addresses the assigned task; Language evaluates grammatical and orthographic quality; and Narrativity evaluates the coherence and cohesion of a source-dependent response.

These dimensions are not fully prompt-invariant. In particular, Content is interpreted according to the requirements of each assignment: it concerns the development of a position in persuasive essays, the relevance and use of information from the supplied text in source-dependent responses, and the development of ideas or events in narrative writing. Likewise, the structural expectations associated with Organization differ between persuasive and narrative writing. Therefore, the trait names should be interpreted according to their prompt-specific scoring guidelines rather than as identical constructs across all prompts.

The original ASAP holistic scores were obtained through human-scoring procedures involving multiple raters, although the score-resolution procedure differed across prompts (Shermis, 2014). Reported human-to-human QWK values range from approximately 0.61 to 0.85. In contrast, the additional ASAP++ trait scores for Prompts 1–6 were generally assigned by a single annotator per essay. A second annotator was used only when an attribute score differed by at least two points from either original overall score, and independent trait-level inter-rater agreement statistics were not reported (Mathias and Bhattacharyya, 2018). This lack of trait-level agreement data is acknowledged as a limitation in Section 6.

The variation in prompt types, scoring ranges, and available traits motivates the unified masked-trait representation used in ARGUE-TIRA. The trait mask ensures that only traits annotated for the corresponding prompt contribute to the training loss, while prompt-specific normalization accounts for differences in scoring ranges. However, the framework does not directly encode the prompt-specific rubric text.

## 4 Methodology

We propose ARGUE-TIRA, a multi-stage framework for joint holistic and analytic automated essay scoring. Unlike conventional multi-task transformer models that map an essay representation directly to multiple prediction heads, ARGUE-TIRA decomposes scoring into three stages: initial trait estimation, trait-interaction reasoning, and holistic decision-making (Figure 1). This design is motivated by rubric-based human assessment, in which analytic dimensions inform the final holistic score.

Given an essay *e*, the objective is to predict a holistic score *y*_*h*_ and an analytic score vector **y**_*t*_ = (*y*_1_, *y*_2_, …, *y*_*T*_), where *T* denotes the number of traits in the unified trait space.

### 4.1 Unified trait space and data processing

To enable joint learning across the heterogeneous prompt-specific rubrics described in Section 3, we define a unified trait space T comprising content, organization, word choice, sentence fluency, conventions, prompt adherence, language, and narrativity, where T=|T| denotes the total number of traits.

Because not all traits are annotated for every prompt, each essay is associated with a binary availability mask **m**∈{0, 1}^*T*^. For trait *j*, *m*_*j*_ = 1 indicates that the trait is available for the essay's prompt, whereas *m*_*j*_ = 0 indicates that it is unavailable. During training, the mask excludes unavailable traits from the analytic scoring loss, allowing the model to learn jointly from all available annotations without treating missing traits as scoring targets.

Because the holistic and analytic scoring ranges vary across prompts and traits, each score is normalized to [0, 1] using the corresponding minimum and maximum values, as shown in Equation 1:


$$\begin{array}{l} ŷ=\frac{y-y_{\min}}{y_{\max}-y_{\min}}, \end{array}$$ (1)

where *y* is the original score and *y*_min_ and *y*_max_ denote the minimum and maximum values defined by the applicable prompt-specific scoring rubric.

### 4.2 Essay representation learning

Each essay is encoded using a pre-trained DistilRoBERTa encoder. DistilRoBERTa was selected for its balance between contextual representation quality and computational efficiency, making it suitable for the proposed multi-task setting. It also achieved the highest average holistic QWK among the evaluated encoder baselines, as reported in Section 5.

For clarity, we omit the batch dimension throughout the following formulation. Given an input token sequence, the encoder produces the contextual token representations defined in Equation 2:


$$\begin{array}{l} \text{H}=\mathrm{Encoder}\left(e\right)\in {\mathbb{R}}^{L\times d}, \end{array}$$ (2)

where *L* denotes the sequence length and *d* denotes the encoder hidden dimension.

A global essay representation is obtained through attention-mask-aware mean pooling, as defined in Equation 3:


$$\begin{array}{l} \text{h}=\frac{\sum_{i=1}^{L}a_{i}{\text{H}}_{i}}{\sum_{i=1}^{L}a_{i}}\in {\mathbb{R}}^{d}, \end{array}$$ (3)

where *a*_*i*_∈{0, 1} denotes the attention-mask value for token *i*, and ${\text{H}}_{i}\in {\mathbb{R}}^{d}$ is its contextual representation. The pooled representation is then projected into a shared latent space, as defined in Equation 4:


$$\begin{array}{l} \text{z}=\mathrm{ReLU}\left(\mathrm{LayerNorm}\left({\text{W}}_{s}\text{h}+{\text{b}}_{s}\right)\right)\in {\mathbb{R}}^{d_{s}}, \end{array}$$ (4)

where the subscript *s* refers to the shared projection layer and *d*_*s*_ denotes the shared representation dimension. The learnable weight matrix and bias vector are ${\text{W}}_{s}\in {\mathbb{R}}^{d_{s}\times d}$ and ${\text{b}}_{s}\in {\mathbb{R}}^{d_{s}}$, respectively.

### 4.3 Architecture

The proposed model consists of three main components: a Trait Estimation Module, a Trait Interaction Reasoning Attention Module, and a Holistic Decision Module.

#### 4.3.1 Trait estimation module

The Trait Estimation Module produces initial analytic trait scores from the shared essay representation, as defined in Equation 5:


$$\begin{array}{l} {\hat{\text{y}}}_{t}^{\left(0\right)}=\sigma \left(f_{\text{trait}}\left(\text{z}\right)\right)\in {\left[0,1\right]}^{T}, \end{array}$$ (5)

where *f*_trait_ is a feed-forward neural network, σ(·) denotes the sigmoid function, and *T* is the number of analytic traits. Thus, ${\hat{\text{y}}}_{t}^{\left(0\right)}$ contains the initial normalized score for each trait.

#### 4.3.2 Trait interaction reasoning attention module

To model dependencies among analytic traits, ARGUE-TIRA introduces the Trait Interaction Reasoning Attention (TIRA) module. TIRA applies self-attention to the initial trait predictions before refining their scores.

As defined in Equation 6, each initial trait score is projected into a trait-level embedding:


$$\begin{array}{l} {\text{r}}_{j}={\text{W}}_{e}{ŷ}_{j}^{\left(0\right)}+{\text{q}}_{j}\in {\mathbb{R}}^{d_{r}}, \end{array}$$ (6)

where *d*_*r*_ denotes the trait-embedding dimension, ${\text{W}}_{e}\in {\mathbb{R}}^{d_{r}\times 1}$ is a learnable projection, and ${\text{q}}_{j}\in {\mathbb{R}}^{d_{r}}$ is a learnable identity embedding for trait *j*. The trait embeddings are stacked to form $\text{R}={\left[{\text{r}}_{1},\ldots ,{\text{r}}_{T}\right]}^{⊤}\in {\mathbb{R}}^{T\times d_{r}}$.

The query, key, and value matrices and the resulting self-attention output are defined in Equation 7:


$$\begin{array}{ll} \text{Q} & =\text{R}{\text{W}}_{Q},\;\text{K}=\text{R}{\text{W}}_{K},\;\text{V}=\text{R}{\text{W}}_{V},\\ {\text{R}}^{\prime } & =\mathrm{MHAttn}\left(\text{Q},\text{K},\text{V}\right)\in {\mathbb{R}}^{T\times d_{r}}, \end{array}$$ (7)

where ${\text{W}}_{Q},{\text{W}}_{K},{\text{W}}_{V}\in {\mathbb{R}}^{d_{r}\times d_{r}}$ are distinct learnable projection matrices, and $\text{Q},\text{K},\text{V}\in {\mathbb{R}}^{T\times d_{r}}$. Thus, **R** supplies the query, key, and value information through three distinct learned projections.

The attention output is subsequently processed using residual connections, layer normalization, and a feed-forward network, as defined in Equations 8, 9:


$$\begin{array}{l} \tilde{\text{R}}=\mathrm{LayerNorm}\left(\text{R}+{\text{R}}^{\prime }\right)\in {\mathbb{R}}^{T\times d_{r}}, \end{array}$$ (8)

$$\begin{array}{l} {\text{R}}^{*}=\mathrm{LayerNorm}\left(\tilde{\text{R}}+\mathrm{FFN}\left(\tilde{\text{R}}\right)\right)\in {\mathbb{R}}^{T\times d_{r}}. \end{array}$$ (9)

Each refined trait representation is then projected back to a scalar update, as defined in Equation 10:


$$\begin{array}{l} \text{Δ}{\text{y}}_{t}=f_{\text{out}}\left({\text{R}}^{*}\right)\in {\mathbb{R}}^{T}. \end{array}$$ (10)

Finally, the refined trait predictions are obtained through residual refinement, as defined in Equation 11:


$$\begin{array}{l} {\hat{\text{y}}}_{t}^{*}=\mathrm{clip}\left({\hat{\text{y}}}_{t}^{\left(0\right)}+\lambda \text{Δ}{\text{y}}_{t},0,1\right)\in {\left[0,1\right]}^{T}, \end{array}$$ (11)

where λ is the refinement weight, set to 0.5 in our implementation. This mechanism enables the model to capture relationships such as content–prompt adherence, organization–sentence fluency, and word choice–language, which are difficult to represent using independent trait heads.

#### 4.3.3 Holistic decision module

The Holistic Decision Module predicts the holistic score by combining the shared essay representation with the refined trait scores, as defined in Equation 12:


$$\begin{array}{l} {ŷ}_{h}=\sigma \left(f_{\text{decision}}\left(\left[\text{z};{\hat{\text{y}}}_{t}^{*}\right]\right)\right)\in \left[0,1\right], \end{array}$$ (12)

where [·;·] denotes concatenation, $\left[\text{z};{\hat{\text{y}}}_{t}^{*}\right]\in {\mathbb{R}}^{d_{s}+T}$, and ŷ_*h*_ is the resulting scalar holistic prediction. Thus, the final decision incorporates both the essay-level representation and the refined analytic trait scores.

### 4.4 Auxiliary holistic and trait-consistency objectives

To stabilize training, an auxiliary holistic prediction is generated directly from the shared essay representation, as defined in Equation 13:


$$\begin{array}{l} {ŷ}_{h}^{\text{aux}}=\sigma \left(f_{\text{aux}}\left(\text{z}\right)\right)\in \left[0,1\right]. \end{array}$$ (13)

A trait-guided holistic prediction is also generated from the refined trait scores, as defined in Equation 14:


$$\begin{array}{l} {ŷ}_{h}^{\text{trait}}=\sigma \left(f_{\text{trait-score}}\left({\hat{\text{y}}}_{t}^{*}\right)\right)\in \left[0,1\right]. \end{array}$$ (14)

Both outputs are scalars. The trait-guided branch encourages the refined analytic scores to remain consistent with the holistic reference score.

### 4.5 Training objective

The model is trained using a multi-task objective that combines final holistic scoring, auxiliary holistic supervision, refined and initial analytic trait prediction, and trait-to-holistic consistency. All loss terms defined below are scalar quantities.

The final holistic loss is defined in Equation 15:


$$\begin{array}{l} L_{h}=SmoothL1\left({ŷ}_{h},y_{h}\right). \end{array}$$ (15)

The auxiliary holistic loss is defined in Equation 16:


$$\begin{array}{l} L_{\text{aux}}=SmoothL1\left({ŷ}_{h}^{\text{aux}},y_{h}\right). \end{array}$$ (16)

The refined analytic trait loss uses a binary mask to exclude unavailable trait annotations, as defined in Equation 17:


$$\begin{array}{l} L_{t}=\frac{\sum_{i=1}^{N}\sum_{j=1}^{T}m_{ij}SmoothL1\left({ŷ}_{ij}^{*},y_{ij}\right)}{\sum_{i=1}^{N}\sum_{j=1}^{T}m_{ij}}. \end{array}$$ (17)

To stabilize the initial trait-estimation stage, the initial predictions are also supervised, as defined in Equation 18:


$$\begin{array}{l} L_{\text{init}}=\frac{\sum_{i=1}^{N}\sum_{j=1}^{T}m_{ij}SmoothL1\left({ŷ}_{ij}^{\left(0\right)},y_{ij}\right)}{\sum_{i=1}^{N}\sum_{j=1}^{T}m_{ij}}. \end{array}$$ (18)

Here, *N* denotes the number of essays considered in the loss computation, *T* denotes the number of analytic traits, and *m*_*ij*_∈{0, 1} indicates whether trait *j* is available for essay *i*.

The trait-to-holistic consistency loss is defined in Equation 19:


$$\begin{array}{l} L_{\text{cons}}=SmoothL1\left({ŷ}_{h}^{\text{trait}},y_{h}\right). \end{array}$$ (19)

The complete optimization objective is defined in Equation 20:


$$\begin{array}{l} L=L_{h}+\alpha L_{\text{aux}}+\beta L_{t}+\gamma L_{\text{init}}+\delta L_{\text{cons}}. \end{array}$$ (20)

The refinement weight λ and the loss weights α, β, γ, and δ were selected empirically based on validation performance and fixed across all experiments. The selected values were λ = 0.5, α = 0.2, β = 0.7, γ = 0.25, and δ = 0.15.

### 4.6 Training configuration

The proposed ARGUE-TIRA framework is trained using the AdamW optimizer with differential learning rates for the transformer backbone and task-specific prediction heads. Specifically, the DistilRoBERTa encoder is fine-tuned using a learning rate of 1 × 10^−5^, while higher learning rates are applied to the reasoning and scoring heads to facilitate faster adaptation. A weight decay of 0.01 is employed for regularization. Essays are tokenized using the DistilRoBERTa tokenizer with a maximum sequence length of 512 tokens to better capture long-range contextual and discourse information.

Gradient accumulation is used for better optimization stability and memory efficiency with an effective batch size of 16 samples, and gradient clipping is used to avoid exploding gradients with a maximum norm of 1.0. The model is trained with a cosine learning rate scheduler and a warm-up period of 10% of the total learning rate steps.

The training objective includes holistic scoring loss, analytic trait prediction loss, auxiliary holistic supervision loss, and consistency regularization between analytic and holistic prediction.

To evaluate the proposed method, the dataset was divided into training, validation, and test splits using stratified sampling based on prompt identifiers to preserve prompt distribution across partitions. The best model was chosen based on the validation loss.

## 5 Results and discussion

### 5.1 Baseline models

To comprehensively evaluate the effectiveness of the proposed ARGUE-TIRA framework, we compare it against a diverse set of AES models spanning traditional neural architectures, transformer-based methods, prompt-aware systems, and recent trait-aware approaches. We consider two evaluation settings: holistic scoring, which predicts a single overall essay score, and analytic scoring, which predicts individual trait scores and reports their average QWK.

- **Hi att** (Dong et al., 2017) is an early hierarchical AES framework that combines CNNs, LSTMs, and attention mechanisms to capture local semantic features, global contextual dependencies, and salient essay content for automated scoring.
- AES aug (Hussein et al., 2020) extends the neural AES model of Taghipour and Ng (2016) into a multi-task framework for simultaneous multi-attribute essay scoring and adaptive feedback generation.
- **PAES** (Ridley et al., 2020) is a prompt-aware AES framework that explicitly incorporates prompt representations into the scoring process. The model jointly learns essay and prompt representations to improve prompt relevance modeling and cross-prompt generalization.
- **CTS** (Ridley et al., 2021) introduces the first cross-prompt multi-attribute AES framework, which employs a multi-task architecture with shared and private layers together with a trait-attention mechanism to model inter-trait relationships and improve cross-prompt trait scoring performance.
- **ProTACT** (Do et al., 2023) is a prompt- and trait-aware framework for cross-prompt essay trait scoring that integrates essay–prompt attention, topic-coherence features, and trait-similarity learning to improve rubric-based scoring performance and interpretability.
- **RDCTS** (Sun et al., 2023) is a cross-prompt trait scoring framework that combines POS and dependency syntax feature fusion with contrastive learning and regularized dropout to improve essay representation, trait-level consistency, model robustness, and scoring stability under limited trait-level training data.
- **DualBERT-Trans-CNN** (Cho et al., 2024) combines hierarchical dual-scale BERT encoders with transformer and convolutional modules to capture global contextual dependencies and local semantic features for joint holistic and multi-trait essay scoring.
- **EPCTS** (Xu et al., 2025) is a prompt-aware cross-prompt trait scoring framework that integrates POS and syntactic embeddings with essay–prompt attention and prompt-relevant semantic features to improve trait-level scoring robustness and generalization under low-resource prompt settings.
- **MOOSE** (Chen et al., 2025) is a cross-prompt multi-trait AES framework that uses a Mixture of Ordered Scoring Experts to improve prompt generalization and trait-aware scoring performance.

In addition to prior studies, we also compare against several internal transformer-based multi-task baselines. **BERT-MT**, **RoBERTa-MT**, and **DistilRoBERTa-MT** are multi-task transformer frameworks that jointly predict holistic and analytic scores using shared contextual representations. These models serve as strong transformer-based baselines for evaluating the effectiveness of trait interaction reasoning.

We further evaluate **ARGUE**, a baseline multi-task reasoning framework without explicit trait-interaction refinement.

The compared approaches employ different learning paradigms and evaluation protocols, as summarized in Table 2. In this study, *single-prompt* models are trained and evaluated within the same prompt distribution, whereas *cross-prompt* models are evaluated on unseen prompts. In contrast, *mixed-prompt* models jointly use essays from multiple prompts in the training, validation, and test sets while preserving prompt proportions.

**Table 2:** Comparison of learning paradigms and training configurations of AES baselines.

Model	Single-prompt	Cross-prompt	Mixed-prompt	MTL	Trait scoring	Prompt-aware	Reasoning-based	Transformer-based
Hi att (Dong et al., 2017)	✓	–	–	–	–	–	–	–
AES aug (Hussein et al., 2020)	✓	–	–	✓	✓	–	–	–
PAES (Ridley et al., 2020)	–	✓	–	–	–	–	–	–
CTS (Ridley et al., 2021)	–	✓	–	✓	✓	–	–	–
ProTACT (Do et al., 2023)	–	✓	–	✓	✓	✓	–	–
RDCTS (Sun et al., 2023)	–	✓	–	✓	✓	–	–	–
DualBERT-Trans-CNN (Cho et al., 2024)	✓	–	–	✓	✓	–	–	✓
EPCTS (Xu et al., 2025)	–	✓	–	✓	✓	✓	–	–
MOOSE (Chen et al., 2025)	–	✓	–	✓	✓	✓	✓	✓
BERT-MT	–	–	✓	✓	✓	–	–	✓
RoBERTa-MT	–	–	✓	✓	✓	–	–	✓
DistilRoBERTa-MT	–	–	✓	✓	✓	–	–	✓
ARGUE	–	–	✓	✓	✓	–	✓	✓
**ARGUE-TIRA (ours)**	–	–	✓	✓	✓	–	✓	✓

Table 3 shows the holistic scoring performance in terms of Quadratic Weighted Kappa (QWK) across various prompts on the ASAP dataset. The results show that ARGUE-TIRA achieves competitive holistic performance relative to the evaluated baselines. Traditional neural models such as Hi-att achieved relatively limited performance, obtaining an average QWK of 0.492, which indicates the limitations of early CNN–LSTM architectures in modeling complex essay characteristics. Prompt-agnostic methods, on the other hand, such as PAES improved the average QWK to 0.654 by incorporating prompt-independent representations and handcrafted linguistic features.

**Table 3:** QWK performance comparison across prompts for the holistic scoring task on ASAP dataset.

Model	P1	P2	P3	P4	P5	P6	Avg.
Hi att (Dong et al., 2017)	0.372	0.465	0.432	0.523	0.586	0.574	0.492
PAES (Ridley et al., 2020)	0.746	0.591	0.608	0.641	0.727	0.609	0.654
PMAES (Chen and Li, 2023)	0.758	0.674	0.658	0.625	0.735	0.578	0.671
BERT-MT	0.740	0.719	0.636	**0.869**	0.797	0.787	0.758
RoBERTa-MT	0.699	0.759	0.618	0.843	0.732	0.798	0.741
DistilRoBERTa-MT	0.746	0.708	0.693	0.866	**0.806**	0.814	**0.772**
ARGUE	0.771	0.755	0.628	0.862	0.762	**0.824**	0.767
**ARGUE-TIRA**	**0.786**	**0.769**	**0.734**	0.786	0.733	0.765	0.762
**Improvement (%)**	+1.95%	+1.32%	+5.92%	–9.55%	–9.06%	–7.16%	–1.30%

Results obtained from Chen and Li (2023). Bold values indicate the best-performing result for each evaluation metric.

Among the transformer-based multitask baselines, DistilRoBERTa-MT achieved the strongest average holistic performance with a QWK of 0.772, followed by ARGUE with 0.767 and BERT-MT with 0.758. Although ARGUE-TIRA achieved the highest performance on Prompts 1, 2, and 3, the overall average QWK was slightly lower than DistilRoBERTa-MT due to reduced performance on Prompts 4–6. This behavior suggests that explicit trait interaction reasoning particularly benefits prompts requiring stronger semantic and organizational reasoning, while certain prompts may favor lighter transformer architectures with stronger generalization characteristics.

Table 4 reports average analytic performance across prompts on ASAP++, while Table 5 presents the trait-level results.

**Table 4:** Average analytic trait scoring performance across prompts on the ASAP++ dataset.

Model	Prompt 1	Prompt 2	Prompt 3	Prompt 4	Prompt 5	Prompt 6	AVG
Hi att (2017) (Dong et al., 2017)	0.315	0.478	0.317	0.478	0.375	0.357	0.387
AES aug (2020)(Hussein et al., 2020)	0.330	0.518	0.299	0.477	0.341	0.399	0.394
PAES (2020) (Ridley et al., 2020)	0.605	0.522	0.575	0.606	0.634	0.545	0.581
CTS no att (2021) (Ridley et al., 2021)	0.619	0.539	0.585	0.616	0.616	0.544	0.589
CTS (2021) (Ridley et al., 2021)	0.623	0.540	0.592	0.623	0.613	0.548	0.590
PMAES (2023) (Chen and Li, 2023)	0.656	0.553	0.598	0.606	0.626	0.572	0.602
ProTACT (2023) (Do et al., 2023)	0.647	0.587	0.623	0.632	0.674	0.584	0.625
RDCTS (2024) (Sun et al., 2023)	0.651	0.553	0.608	0.623	0.651	0.580	0.611
EPCTS (2025) (Xu et al., 2025)	0.659	0.609	0.619	0.686	0.671	0.629	0.646
BERT-MT	0.5923	0.6724	0.6747	0.7953	0.6973	0.7116	0.6906
RoBERTa-MT	0.6334	0.7442	0.7073	**0.8009**	0.6955	**0.7684**	0.7250
DistilRoBERTa-MT	0.5316	0.6675	0.6328	0.7675	0.6977	0.7261	0.6705
ARGUE	0.6405	0.7606	0.7204	0.7820	0.6965	0.7583	0.7264
**ARGUE-TIRA (Ours)**	**0.6667**	**0.7689**	**0.7338**	0.7862	**0.7335**	0.7650	**0.7424**
**Improvement (%)**	+1.17%	+1.09%	+1.86%	–1.84%	+5.13%	–0.44%	+2.20%

Baseline results obtained from Chen and Li (2023) and Ridley et al. (2021). Bold values indicate the best-performing result for each evaluation metric.

**Table 5:** Average QWK scores across all prompts for each trait on the ASAP++ dataset.

Model	Content	Org	WC	SF	Conv	PA	Lang	Nar	Avg
Hi att (Dong et al., 2017)	0.348	0.243	0.416	0.428	0.244	0.309	0.293	0.379	0.346
AES aug (Hussein et al., 2020)	0.342	0.256	0.402	0.432	0.239	0.331	0.313	0.377	0.344
PAES (2020) (Ridley et al., 2020)	0.539	0.414	0.531	0.536	0.367	0.570	0.531	0.605	0.527
CTS (2021) (Ridley et al., 2021)	0.555	0.458	0.557	0.545	0.412	0.565	0.536	0.608	0.586
CTS no att (2021) (Ridley et al., 2021)	0.541	0.424	0.558	0.544	0.387	0.561	0.539	0.605	0.535
PMAES (2023) (Chen and Li, 2023)	0.567	0.481	0.584	0.582	0.421	0.584	0.545	0.614	0.561
ProTACT (2023) (Do et al., 2023)	0.596	0.518	0.599	0.585	0.450	0.619	0.596	0.639	0.586
RDCTS (2024) (Sun et al., 2023)	0.561	0.480	0.591	0.576	0.426	0.609	0.560	0.634	0.568
DualBERT-Trans-CNN (2024) (Cho et al., 2024)	0.726	0.682	0.680	0.663	0.668	0.732	0.660	0.704	0.683
EPCTS (2025) (Xu et al., 2025)	0.630	0.606	0.614	0.617	0.525	0.630	0.613	0.647	0.623
MOOSE (2025) (Chen et al., 2025)	0.651	0.652	0.634	0.643	0.604	0.649	0.624	0.665	0.641
BERT-MT	0.7161	0.5947	0.6553	0.6196	0.6353	0.7453	0.6692	0.7186	0.6693
RoBERTa-MT	0.7488	0.6640	0.6874	0.6954	0.6883	0.7487	0.6993	**0.7554**	0.7109
DistilRoBERTa-MT	0.7070	0.5703	0.6208	0.5884	0.5914	0.7313	0.6482	0.6976	0.6444
ARGUE	0.7450	0.6787	0.7054	0.7070	0.6960	0.7496	0.7142	0.7337	0.7162
**ARGUE-TIRA (Ours)**	**0.762**	**0.703**	**0.724**	**0.720**	**0.719**	**0.761**	**0.731**	0.745	**0.733**
**Improvement (%)**	+1.76%	+3.08%	+2.64%	+3.54%	+3.05%	+1.51%	+2.35%	–1.34%	+3.13%

Results obtained from Chen and Li (2023) and Ridley et al. (2021). The best performance for each trait is highlighted in bold.

Org, Organization; WC, Word Choice; SF, Sentence Fluency; Conv, Conventions; PA, Prompt Adherence; Lang, Language; Nar, Narrativity. Improvement is computed relative to the strongest previously reported baseline for each trait.

For average analytic trait scoring, ARGUE-TIRA achieves the highest overall average QWK score of 0.7424 across the evaluated prompts, outperforming all competing methods included in the comparison. Compared with the strongest prior baseline, ARGUE, which achieved an average QWK of 0.7264, ARGUE-TIRA improves the overall average performance by approximately 2.20%. The proposed framework achieves the best performance on Prompt 1 (0.6667), Prompt 2 (0.7689), Prompt 3 (0.7338), and Prompt 5 (0.7335). Although RoBERTa-MT achieves slightly higher performance on Prompt 4 (0.8009) and Prompt 6 (0.7684), ARGUE-TIRA remains highly competitive while providing the strongest overall average performance across prompts. These results indicate that the proposed reasoning-based framework generalizes effectively across prompts with heterogeneous rubric structures and varying writing styles.

The results further reveal substantial variability across prompts, highlighting the inherent difficulty differences among essay topics and scoring rubrics. In particular, Prompt 1 consistently achieves comparatively lower QWK scores across most models in both holistic and analytic evaluation settings, indicating that it is among the more challenging prompts for automated essay scoring systems. Prompt 1 involves highly subjective narrative writing, where students describe personal experiences using diverse storytelling styles, implicit discourse progression, and highly variable semantic structures. This variability reduces structural regularity between essays and weakens the effectiveness of surface-level linguistic patterns. Prompt 3 also demonstrates relatively lower performance in several baseline methods, suggesting additional challenges related to semantic diversity and rubric variability. Prompt 5 remains comparatively challenging in some holistic evaluation settings due to substantial variability in argument quality, coherence, and linguistic expression.

In contrast, Prompts 4 and 6 show stronger and more stable performance across transformer-based approaches. Their structured argumentative or expository responses exhibit clearer organization, stronger lexical regularities, and closer alignment with rubric expectations, making them easier for the models to learn.

Another factor is the rubric structure that is heterogeneous in the ASAP/ASAP++ datasets. The different prompts emphasize different dimensions of scoring such as organization, relevance of content, creativity, or language quality. Consequently, the importance of specific analytic traits varies significantly across prompts. Narrative prompts may be more focused on coherence, creativity, and tell-ability of the writing, whereas argumentative and expository prompts place stronger emphasis on the organization of evidence and linguistic consistency and logical structure of the writing.

These findings suggest that TIRA's explicit modeling of inter-trait dependencies helps the framework accommodate heterogeneous rubric structures and produce more consistent scores across prompts.

The results in Table 5 for the analytic traits further demonstrate the effectiveness of the proposed reasoning framework. ARGUE-TIRA achieves the highest performance in seven out of eight analytic traits, including Content (0.762), Organization (0.703), Word Choice (0.724), Sentence Fluency (0.720), Conventions (0.719), Prompt Adherence (0.761), and Language (0.731). Although RoBERTa-MT achieves slightly higher performance on the Narrativity trait (0.755), ARGUE-TIRA remains highly competitive with a score of 0.745 while achieving the highest overall average analytic QWK score of 0.733. Compared with the strongest competing baseline, ARGUE, which achieved an average score of 0.716, the proposed framework demonstrates consistent improvements across most rubric dimensions.

Noticeable gains are observed for Organization, Sentence Fluency, Conventions, and Language, suggesting that inter-trait modeling helps capture discourse coherence, grammatical quality, and linguistic fluency. Strong performance on Prompt Adherence and Content further indicates alignment with prompt-specific semantic requirements.

Although ARGUE-TIRA achieves strong overall analytic performance, Narrativity remains more challenging than the other rubric dimensions. This trait depends on storytelling quality, narrative coherence, emotional progression, and event consistency, which require high-level discourse understanding and long-range semantic reasoning. Essays of similar narrative quality may also use different vocabulary, styles, and discourse structures, limiting the effectiveness of surface-level textual similarity.

Although self-attention improves contextual representation learning, compressing lengthy essays into a single representation may dilute important discourse-level signals.

ARGUE-TIRA partially addresses these limitations by modeling complementary relationships among Organization, Narrativity, Content, and Sentence Fluency. Nevertheless, the comparatively lower Narrativity score indicates that discourse-level reasoning and narrative understanding remain open challenges.

The results also confirm the advantage of transformer-based contextual representations over earlier neural architectures. ARGUE-TIRA extends this advantage through an intermediate stage that explicitly models interactions among analytic traits.

Overall, the results demonstrate that trait-interaction reasoning substantially improves analytic scoring while maintaining competitive holistic performance. The framework achieves the highest average analytic QWK and the best results in seven of the eight evaluated traits.

As discussed in Section 4, these comparisons should be interpreted in relation to the reliability of the human-assigned reference scores.

## 6 Limitations

Several limitations should be considered when interpreting the results. First, automated scoring performance depends on the reliability of the reference annotations. Although the original ASAP holistic scores were produced through human-scoring procedures involving multiple raters, human-to-human QWK varies across prompts, with reported values ranging from approximately 0.61 to 0.85 (Shermis, 2014). This variation indicates that the consistency of human scoring depends on the writing task, rubric, and score-resolution procedure. Therefore, model QWK should be interpreted in relation to the reliability of the corresponding human scores.

Second, the additional ASAP++ analytic annotations for Prompts 1–6 were generally assigned by a single annotator per essay (Mathias and Bhattacharyya, 2018). A second annotator was involved only when an attribute score differed by at least two points from either of the original overall scores. Because independent inter-rater agreement statistics were not reported for these trait annotations, the reliability ceiling for analytic scoring cannot be estimated. The reported trait-level QWK values consequently measure agreement with the released annotations rather than with a fully adjudicated multi-rater gold standard.

Third, although ARGUE-TIRA achieves the highest average analytic QWK and the best results for seven of the eight evaluated traits, its improvement over the strongest comparison model is modest. The average prompt-level analytic QWK increases from 0.7264 for ARGUE to 0.7424 for ARGUE-TIRA, representing an absolute gain of 0.016 and a relative improvement of approximately 2.20%. The gains for individual traits are also generally small. These findings support the potential value of explicit trait-interaction modeling but do not establish a large practical improvement, particularly in the absence of trait-level human agreement estimates and statistical significance tests against all published baselines.

Fourth, although the complete ASAP/ASAP++ resource contains eight prompts representing three writing conditions, the present experiments use only Prompts 1–6, which cover persuasive and source-dependent writing. Moreover, the definitions and scoring criteria of traits such as Content and Organization vary across prompts. The proposed masking and normalization procedures accommodate differences in trait availability and scoring ranges, but the model does not explicitly encode the complete prompt-specific scoring guidelines. The learned trait interactions may therefore partly reflect the particular rubric structures and score distributions of the evaluated ASAP++ prompts.

Finally, the experiments are limited to English essays from a single benchmark family. Evaluation on independently collected datasets, additional educational levels, other writing genres, and multilingual essays is necessary before conclusions can be drawn regarding broader generalizability or deployment in educational assessment. Future work should therefore examine multi-rater analytic datasets, uncertainty-aware scoring, rubric-conditioned models, and direct comparisons with human-level agreement.

## 7 Conclusion

This paper introduces ARGUE-TIRA, a multi-task framework that explicitly models interactions among analytic writing traits for joint holistic and analytic essay scoring. The proposed framework captures dependencies among analytic rubric dimensions before holistic score prediction, thereby providing a more structured and interpretable scoring process. Experimental results on the ASAP and ASAP++ datasets demonstrate that ARGUE-TIRA achieves competitive holistic performance and superior analytic trait-scoring performance, attaining the highest average analytic QWK and the best results in seven of the eight evaluated traits.

In particular, the results demonstrate that trait-interaction modeling contributes to capturing discourse-level coherence, language quality, sentence fluency, and prompt adherence across heterogeneous prompt-specific rubrics. Although Narrativity remains comparatively challenging because it requires high-level discourse understanding and the modeling of long-range semantic relationships, the proposed framework improves analytic trait-scoring performance while maintaining competitive holistic performance.

Overall, the findings indicate that explicitly modeling dependencies among analytic writing dimensions is a promising direction for improving the accuracy and interpretability of automated essay scoring. Future work will explore stronger discourse modeling, adaptive prompt-aware mechanisms, and large language model integration to enhance scoring robustness and generalizability.

## Data Availability

Publicly available datasets were analyzed in this study. This data can be found here: https://lwsam.github.io/ASAP++/lrec2018.html.
